# Effects of massage therapy and occlusal splint therapy on electromyographic activity and the intensity of signs and symptoms in individuals with temporomandibular disorder and sleep bruxism: a randomized clinical trial

**DOI:** 10.1186/s12998-014-0043-6

**Published:** 2014-12-15

**Authors:** Cid André Fidelis de Paula Gomes, Yasmin El Hage, Ana Paula Amaral, Fabiano Politti, Daniela Aparecida Biasotto-Gonzalez

**Affiliations:** Postgraduate Program in Biophotonics Applied to Health Sciences, Universidade Nove de Julho (UNINOVE), Av. Dr. Adolfo Pinto,109, Água Branca, 05001-100 São Paulo, SP Brazil; Postgraduate Program in Rehabilitation Sciences, Universidade Nove de Julho (UNINOVE), Av. Dr. Adolfo Pinto,109, Água Branca, 05001-100 São Paulo, SP Brazil; Department of Physical Therapy, Universidade Nove de Julho (UNINOVE), Av. Dr. Adolfo Pinto,109, Água Branca, 05001-100 São Paulo, SP Brazil

**Keywords:** Physical therapy modalities, Temporomandibular joint disorders, Massage therapy, Occlusal splint

## Abstract

**Introduction:**

Temporomandibular disorder (TDM) is the most common source of orofacial pain of a non-dental origin. Sleep bruxism is characterized by clenching and/or grinding the teeth during sleep and is involved in the perpetuation of TMD. The aim of the present study was to investigate the effects of massage therapy, conventional occlusal splint therapy and silicone occlusal splint therapy on electromyographic activity in the masseter and anterior temporal muscles and the intensity of signs and symptoms in individuals with severe TMD and sleep bruxism.

**Methods:**

Sixty individuals with severe TMD and sleep bruxism were randomly distributed into four treatment groups: 1) massage group, 2) conventional occlusal splint group, 3) massage + conventional occlusal splint group and 4) silicone occlusal splint group. Block randomization was employed and sealed opaque envelopes were used to conceal the allocation. Groups 2, 3 and 4 wore an occlusal splint for four weeks. Groups 1 and 3 received three weekly massage sessions for four weeks. All groups were evaluated before and after treatment through electromyographic analysis of the masseter and anterior temporal muscles and the Fonseca Patient History Index. The Wilcoxon test was used to compare the effects of the different treatments and repeated-measures ANOVA was used to determine the intensity of TMD.

**Results:**

The inter-group analysis of variance revealed no statistically significant differences in median frequency among the groups prior to treatment. In the intra-group analysis, no statistically significant differences were found between pre-treatment and post-treatment evaluations in any of the groups. Group 3 demonstrated a greater improvement in the intensity of TMD in comparison to the other groups.

**Conclusion:**

Massage therapy and the use of an occlusal splint had no significant influence on electromyographic activity of the masseter or anterior temporal muscles. However, the combination of therapies led to a reduction in the intensity of signs and symptoms among individuals with severe TMD and sleep bruxism.

**Trial registration:**

This study is registered in August, 2014 in the ClinicalTrials.gov (NCT01874041).

**Electronic supplementary material:**

The online version of this article (doi:10.1186/s12998-014-0043-6) contains supplementary material, which is available to authorized users.

## Background

Temporomandibular disorder (TMD) is considered the most common source of orofacial pain of a non-dental origin and constitutes a heterogeneous group of conditions that affect the temporomandibular joint and/or masticatory muscles [1]. Sleep bruxism is characterized by clenching and/or grinding one’s teeth during sleep and may be involved in triggering and/or perpetuating TMD [2]. Treatment strategies for both conditions are founded on minimally invasive, reversible interventions involving a multidisciplinary team [3].

In recent years, physiotherapists have employed different resources, such as electrotherapy [4], phototherapy [5], mobilization [6] and massage therapy [7,8,3], with the aim of improving symptoms related to TMD and sleep bruxism through the reestablishment of local blood flow and consequent improvement in muscle function and local analgesia [9]. In dentistry, occlusal splint therapy has been used for this purpose and is founded on the notion that a bite plate promotes relaxation of the masticatory muscles, protects the teeth and jaws from the adverse effects of bruxism, normalizes proprioception of the periodontal ligament and repositions the condyles and jaws into their correct alignment (centric relation) [10].

Despite the large number of randomized clinical trials addressing TMD and sleep bruxism, systematic reviews of the literature [7,11] point out the need for further investigations due to the low degree of methodological quality in previously published papers, such as failures regarding blinding and the use of reliable measures. Moreover, the multifactor etiology of both TMD and sleep bruxism suggests that it may be advantageous to investigate the effects of different resources used either alone or in combination.

The aim of the present study was to investigate the effects of massage therapy, traditional occlusal splint therapy and silicone occlusal splint therapy on electromyographic activity in the masseter and anterior temporal muscles and the intensity of signs of symptoms in individuals with severe TMD and sleep bruxism. The hypothesis tested was that the combination of massage therapy and a muscle-relaxing oral appliance would lead to a decrease electromyographic activity in the masticatory muscles as well as a reduction in the intensity of signs and symptoms of TMD.

## Methods

### Subjects

A blinded, randomized, clinical trial was conducted. The procedures of the present study received approval from the Human Research Ethics Committee of Nove de Julho University, Sao Paulo, Brazil (protocol number 133012). This study is registered in August, 2014 in the ClinicalTrials.gov (NCT01874041). Prior to participation, all volunteers signed a statement of informed consent.

Individuals diagnosed with TMD and sleep bruxism aged 18 to 40 years were recruited from the university community of the city of Sao Paulo, Brazil, through notices placed on information boards located in general areas of the university and the Internet between June 2011 and December 2012. Neither the examiner in charge of the surface electromyography (EMG) nor the researcher in charge of the data analysis was aware of the allocation of the volunteers to the different groups.

The volunteers received an intra-oral examination by an experienced dentist for signs of sleep bruxism. Those with incisal and/or occlusal tooth wear and clinical signs in the buccal mucosa and tongue of clenching or grinding were diagnosed with bruxism based on the criteria of the American Academy of Sleep Medicine [12] and a positive self-report of awake bruxism [13].

The Fonseca Patient History Index was used to diagnose the presence and intensity of TMD, which is an adequately valid, reliable measure for identifying individuals with TMD that has been widely used in recent studies [14-18]. This Portuguese-language measure for the assessment of TMD severity has 10 items, each with three response options: “yes” (scored as 10 points), “sometimes” (5 points) and “no” (0 points). The sum of the points for all items gives the overall score and allows the following classification: absence of TMD (0 to 15 points), mild TMD (20 to 45 points), moderate TMD (50 to 65 points), and severe TMD (70 to 100 points) [18]. Only individuals with severe TMD and bruxism for at least one year were included in the present study.

The following were the exclusion criteria: occurrence of missing teeth (except third molars); current use of an orthodontic appliance; history of neuromuscular disease; current use of analgesic, anti-inflammatory agent or muscle relaxant; and currently undergoing physical therapy for TMD.

One hundred nine male and female individuals were consecutively recruited and screened based on the eligibility criteria. During this process, 49 volunteers were excluded. Thus, the final sample was made up of 60 volunteers with severe TMD and sleep bruxism. Block randomization was employed and opaque envelopes were used to conceal the allocation of the volunteers to the four treatment groups described below (Additional file 1: Figure S1).

The massage group (MG) (n =15; 13 women and 2 men; mean age: 29.32 ± 4.31 years) was submitted to three weekly 30-minute sessions of massage therapy performed by a physiotherapist who had undergone a training exercise for the administration of sliding and kneading maneuvers of the masseter and anterior temporal muscles, bilaterally, over four consecutive weeks (total: 12 sessions) [19]. Sliding consisted of a unidirectional movement in which part of the therapist’s hand (mainly the fingertips) was used, moving from the proximal to the distal portion of the face with constant, progressive pressure compatible with the status of each tissue. The degree of pressure varied depending on the level of pain, sensitivity and tension in each individual. Kneading consisted of a gripping maneuver of a muscle group or portion of a muscle, with intermittent movements of compression and decompression. The therapist performed circular movements with the fingertips such that the skin and subcutaneous tissues were moved over the subjacent structures. A facial massage cream was used to facilitate the manual procedures [15].

The conventional occlusal splint group (COSG) (n =15; 12 women and 3 men; mean age: 27.89 ± 5.82 years) was submitted to treatment with an occlusal splint for four weeks. The aim of this form of treatment was to promote greater stability of the joint components, establish a more favorable occlusal relationship, promote the reorganization of neuromuscular activity, reduce hyperactivity of the masticatory muscles and reestablish balanced muscle function. After a clinical examination by a dentist, the upper arch of each volunteer was molded with irreversible hydrocolloid for the fabrication of a Michigan-type occlusal splint with canine and protrusive guides as well as a flat occlusal surface for contact with the antagonist teeth. The volunteers were instructed to wear the splint while sleeping. Adjustments were made after two weeks by the same dentist in charge of the evaluation and splint fabrication [20,21,15].

The massage + conventional occlusal splint group (MCOSG) (n = 15; 14 women and 1 man; mean age: 26.05 ± 3.32 years) was submitted to combined treatment with massage and occlusal splint as described in the previous two paragraphs.

The silicone occlusal splint group (SOSG) (n = 15; 10 women and 4 men; mean age: 28.92 ± 6.78 years) was submitted to treatment with a silicone splint for four weeks fabricated from a 3-mm soft polyvinyl sheet in a vacuum pressure molding device with a thermally controlled infrared heater. This machine performed vacuum suctioning of the warmed sheet of thick, resilient mouth-guard material over the maxillary cast. When the sheet had properly adapted to the cast in the vacuum former, the splint was separated from the cast with a laboratory knife/scissors, the edges were smoothed and the palatal area was removed. Chair-side occlusal fitting was made by evenly warming the occlusal surface of the splint with an alcohol torch before placement in the patient’s mouth. The soft splint was then polished with pumice, disinfected and placed in the oral cavity. The soft, resilient material is believed to help distribute the load during parafunctional activity (bruxism) [22]. The volunteers were instructed to wear the splint while sleeping.

### Electromyographic analysis

The right and left masseter and anterior temporal muscles were analyzed using surface EMG. EMG signals were obtained using an eight-channel module (EMG System do Brasil Ltda®) consisting of conditioner with a band pass filter with cut-off frequencies at 20–1000 Hz, an amplifier gain of 1000 and a common mode rejection ratio >120 dB. All data were acquired and processed using a 16-bit analog to digital converter (EMG System do Brasil Ltda®) with a sampling frequency 2 kHz. The system was composed of active bipolar electrodes with a pre-amplification gain of 20 x.

The volunteer was instructed to remain seated in a chair, feet apart, shoulders relaxed and hands resting on thighs, with the head on the Frankfurt parallel to the ground and no visual feedback of the signals registered on the computer. The sites for the electrodes were cleaned with a cotton ball soaked in alcohol to diminish impedance between the skin and electrodes. Disposable circular electrodes (Ag/AgCl – Medical Trace^©^) measuring 10 mm in diameter were attached to the belly of the muscle in the region with the greatest tonus after the volunteer performed moderate clenching. Bandage tape was used to secure the electrodes further, with care taken to avoid micro-movements. The inter-electrode distance was 20 mm from center to center, as suggested by the European Recommendations for Surface Electromyography [23]. A rectangular metallic electrode measuring 3 x 2 cm coated with Lectron II conductive gel (Pharmaceutical Innovations®) to increase the conduction capacity and impede interference from external noise was attached to the left wrist of the volunteers for reference [23].

Three EMG readings were taken before and after treatment in all groups during maximum voluntary clenching for 10 seconds, with a five-minute rest period between readings. To avoid direct occlusal contact between the dental cusps, a strip of Parafilm M® (American National Can TM, Chicago, USA) with texture and dimensions similar to commercial chewing gum was folded into five parts and positioned in the molar region bilaterally.

The initial two seconds and final three seconds of the EMG signal were discarded. Thus, a five-second reading was considered for each test. The signal was analyzed using Fast Fourier Transform computed with a 2048-point hamming window processing (50% overlap). The median frequency (MDF) of the power spectrum was calculated for each five-second period analyzed. All EMG signal processing was performed using specific routines carried out in the Matlab program, version 7.1 (The MathWorks Inc., Natick, Massachusetts, USA).

### Data analysis

The sample size was calculated with the aid of the Ene software, version 3.0 (Barcelona, Spain) and based on the findings of a pilot study. The data were collected during maximum voluntary clenching for eight seconds before (T0) and after five sessions of massage therapy (T1). Considering an 80% test power and α of 0.05, the suggested sample size was 13 individuals (T0 = 1.19; T1 = 1.08; SD =0.14) per group.

As the Shapiro-Wilk test revealed that the MDF was not normally distributed (p <0.05), the data were analyzed using nonparametric tests and expressed as median and inter-quartile range (25% and 75%). The Kruskal-Wallis test used to determine the similarity in the MDF among the groups (MG, COSG, MCOSG, SOSG) before treatment (baseline). The Wilcoxon test was used to evaluate the effects of the treatments. TMD intensity before and after treatment was determined using the Fonseca Patient History Index and verified through repeated-measures analysis of variance (ANOVA) considering two factors: group (MG vs. COSG vs. MCOSG vs. SOSG) and treatment (pre-treatment vs. post-treatment). Specific differences were analyzed using Tukey’s post hoc test. The significance level was set to 5% (p <0.05). All data were analyzed using the Statistical Package for the Social Sciences, version 17.

## Results

**Figure 1:**
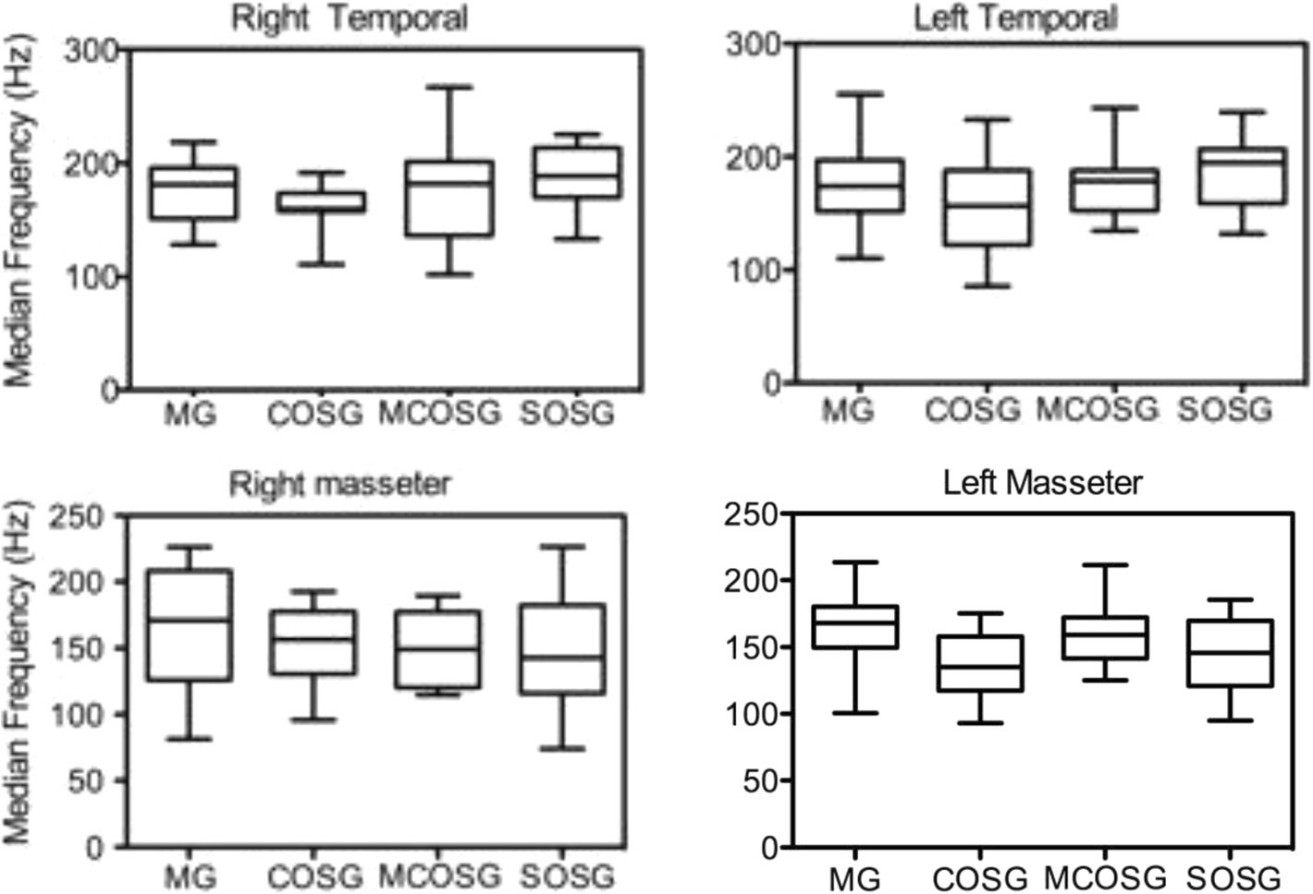
**Median (25% and 75% quartile) of median frequency (Hz) in right and left anterior temporal and masseter muscles during maximum voluntary clenching in each group (non-significant differences,** ***p*** **> 0.05; Kruskal-Wallis test).**

Inter-group analysis of variance revealed no statistically significant differences in the MDF among the MG, COSG, MCOSG and SOSG prior to treatment, thereby excluding the possibility that any group was more or less susceptible to the influence of the treatment employed (Figure 1).

In the intra-group analysis, no statistically significant differences were found between the pre-treatment and post-treatment evaluations of the left and right masseter and anterior temporal muscles in any group (Table 1).

**Table 1:** **Pre-treatment and post-treatment MDF in right temporal (RT), right masseter (RM), left temporal (LT) and left masseter (LM) during maximum voluntary clenching in different groups (Wilcoxon signed rank test)**

		**MG**	**COSG**	**MCOSG**	**SOSG**
**RT**	**Pre**	181.9 [136.2-201.4]	188.7 [169.9-213.6]	189,5 [140.9-203.1]	159.7 [157.7-173.6]
	**Post**	173.1 [133.3-189.5]	195.8 [182.9-210.1]	198.0 [138.4-206.1]	168.7 [156.5-180.2]
	***p***	0.65	0.69	0.73	0.44
**RM**	**Pre**	170.9 [150.9-185.5]	151.6 [116.9-168,9]	142.8 [116.8-182.1]	156.5 [130.6-177.5]
	**Post**	155.3 [113.3-187.3]	160.4 [135.7-183,8]	149.2 [120.4-177.2]	148.7 [121.3-167.5]
	***p***	0.22	1.00	0.86	0.91
**LT**	**Pre**	178.7 [152.3-188.2]	194.8 [158.9-206.8]	176.1 [143.6-193.8]	156.7 [122.1-188.2]
	**Post**	168.7 [147.2-199.7]	193.4 [158.4-206.3]	183.4 [136.4-196.9]	170.4 [145.5-208.7]
	***p***	0.82	0.91	0.36	0.60
**LM**	**Pre**	157.5 [140,6-169.4]	145.8 [120.8-169.4]	142.1 [118.2-171.6]	135.0 [117.4-158.0]
	**Post**	142.3 [123.5-170.4]	154.3 [122.3-179.7]	152.6 [124.3-157.5]	143.8 [90.09-155.8]
	***p***	0.60	0.67	0.82	0.15

MG: massage therapy group; COSG: conventional occlusal splint group; MCOSG: massage therapy + conventional occlusal splint group; SOSG: silicone occlusal splint group.

Table 2 displays the results of the group-treatment interactions regarding TMD intensity based on the Fonseca Patient History Index. Significant differences were found between the pre-treatment and post-treatment evaluations in the MG, COSG and MCOSG, the latter of which exhibited greater improvement in comparison to the other groups.

**Table 2:** **Mean e standard deviation and the results of interactions between groups (ANOVA) pre e post treatment relative intensity according to Fonseca Patient History Index**

	**Treatment (Mean ± SD)**		**ANOVA**		
**Groups**	**Pre**	**Post**	**Interactions**	**F**	**P**
MG	67±12,5	50,33 ±10,76^a^	Treatment (Pré x Post)	32,41	<0,0001
COSG	52 ±15,44	44 ±13,78^a^	Groups (MG x COSG x MCOSG x SOSG)	8,88	<0,0001
MCOSG	54,33±21,03	29,33±10,83^a,b^			
SOSG	56±16,16	59,33±13,21			

^a^Significant difference between pre e post treatment (ANOVA, Bonferroni corrections).

^b^Significant difference between groups MCOSG > MG, COSG, SOSG (Test post hoc de Tukey).

## Discussion

The lack of significant differences in the MDF during the pre-treatment evaluation demonstrated the similarity among the groups at baseline. Moreover, no statistically significant reductions in EMG activity were found in any of the groups after treatment, even when treatment involved the combination of massage and occlusal splint therapy. These findings are in agreement with data reported in previous studies on massage therapy [24-26] and occlusal splint therapy [27,28], which also found no reduction in EMG activity among individuals with TMD.

Hyperactivity of the masseter muscles and constant activity of the anterior temporal muscles even when the mandible is in the resting position is a common characteristic among individuals with sleep bruxism and TMD [29]. Such individuals exhibit reduced blood flow to the masticatory muscles due to vasoconstriction stemming from muscle hyperactivity, which impedes the transport of nutrients and metabolites and can cause the buildup of by-products, thereby triggering pain [30].

Massage therapy has been shown to stimulate parasympathetic activity by engaging the relaxation response, which, in turn, reduces hormonal and autonomic indicators and has an analgesic effect caused by the activation of a pain-gate mechanism, which disables pain signals travelling to central nervous system through larger, faster conductive nerve fibers [9,29,31,32]. Thus, massage can lead to an increase in mandibular function [15] as well as a reduction in the frequency and intensity of bruxism and symptoms of TMD [33]. However, contrary to expectations, massage therapy, occlusal splint therapy or a combination of the two was insufficient to reduce muscle activity in the present study, as demonstrated by the analysis of the masseter and anterior temporal muscles. This may have been related to the severe, chronic characteristics of the participants in the present study, who had sleep bruxism and TMD for at least five years.

The use of occlusal splints for individuals with bruxism and TMD is a common practice in dentistry. However, the mechanism of action has not yet been fully clarified. Indeed, an occlusal splint does not cure TMD or sleep bruxism, but can contribute to improving quality of life [34], increasing maximum mouth opening [22] and reducing myofascial pain [28]. A number of hypotheses may explain the mechanism of action of this type of treatment: 1) a reduction in EMG activity of the masticatory muscles (which did not occur in the present study); 2) the repositioning of the condyle and/or joint disc; 3) a change in the occlusion; and 4) a change in oral habits [35].

Despite being one of the hypotheses for the function of occlusal splint therapy, considerable variation is found in the literature regarding the reduction in EMG activity of the masticatory muscles. Some studies indicate only immediate reductions in EMG activity after the use of an occlusal splint, whereas no reductions occur after long-term use of an occlusal splint [25,26]. These findings suggest the effect of occlusal splints, including silicone splints indicated to control parafunctional activity, is not maintained for a long time due to the adaptive mechanisms of the muscles. Thus, the benefits seem to be temporary and non-cumulative and continual daily use seems to be required to maintain the effect, which may explain the present results, as evaluations were only performed before and after four weeks of occlusal splint use.

TMD and sleep bruxism treatment should be performed by a multidisciplinary team [36] involving a combination of therapeutic resources. The present study involved both massage therapy and occlusal therapy. However, the findings demonstrated no changes in EMG activity among individuals with a diagnosis of chronic, severe TMD and sleep bruxism. Thus, EMG activity is not the best variable for the evaluation of improvements in such individuals and preference should be given to clinical characteristics, such as pain and vertical mandibular movement. Indeed, massage therapy is reported to achieve positive results regarding a reduction in pain and an increase in mandibular function.

Occlusal splint therapy can lead to a reduction in fatigue of the masticatory muscles, as demonstrated in a study by Zhang et al. [37], who found EMG changes in the masticatory muscles following occlusal splint therapy employed for the same period of time as that used in the present study. However, the individuals in the present investigation had a greater intensity and longer duration of signs and symptoms of TMD, which may explain the lack of EMG changes in the sample.

Among individuals with TMD and bruxism, surface EMG is fundamentally employed to demonstrate dysfunction in the masticatory muscles. However, divergent opinions are found regarding its use as a diagnostic tool and parameter of clinical improvement [38]. Moreover, a weak correlation is reported between symptoms of TMD, such as pain, and electrical activity in the masticatory muscles [39], which makes EMG questionable when used as the main reference for the determination of clinical improvement, especially in individuals with signs and symptoms of severe TMD.

The present results regarding the intensity of TMD following the different forms of therapy are in agreement with data reported in a previous study [37] and underscore the importance of the clinical history and physical exam based on signs and symptoms. The improvement found when massage therapy and occusal splint therapy were combined indicates that this combination of treatment modalities is highly desirable when there is a need to diminish clinical signs and symptoms, such as pain in the masticatory muscles, when treating individuals with chronic characteristics associated with sleep bruxism and severe mandibular limitations, as found in the present sample.

There are a number of limitations in the present study that should be recognized. A convenience sample was used and was limited to the university community, which may not be representative of the entire population of individuals with bruxism and TMD. Due to the demand and need for urgent care for the individuals who seek our laboratory, which is a reference service for the treatment of TMD and sleep bruxism, it was not possible to form a control group in an attempt to structure this group with the formation of a silicone occlusal splint, which is a procedure used only for emergencies or as a temporary measure until a conventional occlusal splint can be fabricated. Moreover, only the long-term results of massage therapy and occlusal therapy were investigated. The massage therapy protocol did not involve intraoral (pterygoid) massage, which may have exerted a direct influence on the findings. Considering the improvement in signs and symptoms of TMD reported in a recent study [3], further studies involving the release of intraoral structures are suggested. Moreover, due to the variations in the effects of different therapies on EMG activity, future studies should be carried out to compare immediate and long-term effects.

## Conclusion

In summary, massage therapy and the use of an occlusal splint did not lead to statistically significant changes in electromyographic activity in the masseter and anterior temporal muscles. However, the combination of therapies led to a reduction in the intensity of signs and symptoms among individuals with severe TMD and sleep bruxism.

## Additional file

Additional file 1: Figure S1 Flowchart of the study.

## Abbreviations

TMD: Temporomandibular disorder
EMG: Electromyography
MG: Massage group
COSG: Conventional occlusal splint group
MCOSG: Massage + conventional occlusal splint group
SOSG: Silicone occlusal splint group
MDF: Median frequency
