# Supplementary material for: Effects of massage therapy and occlusal splint therapy on electromyographic activity and the intensity of signs and symptoms in individuals with temporomandibular disorder and sleep bruxism: a randomized clinical trial
Source: Chiropr Man Therap. 2014 Dec 15;22:43. doi: 10.1186/s12998-014-0043-6 (PMC4266206; doi:10.1186/s12998-014-0043-6)
Supplement: Additional file 1: Figure S1 — Flowchart of the study. [file 12998_2014_43_MOESM1_ESM.docx]

Not eligible (n = 49)

Subjects screened for eligibility (n = 109)

Eligible (n = 60)

TMD level < severe (n = 25)

In orthodontic treatment (n = 16)

Toothloss (n = 6)

Use of drugs (n = 2)

Randomization (n = 60)

Participated in the statistical analysis (n = 15)

Participated in the statistical analysis (n = 15)

Participated in the statistical analysis (n = 15)

Participated in the statistical analysis (n = 15)

Dropped out (n = 0)

Dropped out (n = 0)

Received the procedure

(n = 15)

Received the procedure

(n = 15)

Allocated to the COSG

(n = 15)

Allocated to the MG

(n = 15)

Allocated to the MCOSG

(n = 15)

Allocated to the SOSG

(n = 15)

Received the procedure

(n = 15)

Received the procedure

(n = 15)

Dropped out (n = 0)

Dropped out (n = 0)

Additional file 1: Figure S1. Flowchart of the study.
